# Establishment and Characterization of a Swim Bladder-Derived Cell Line from Chu’s Croaker (*Nibea coibor*) for Studying Collagen Regulation

**DOI:** 10.3390/ani16203200

**Published:** 2026-10-12

**Authors:** Yuhong Lin, Yuping Wang, Qi Pan, Yuying Yuan, Xiaobo Wen, Fan Lin

**Affiliations:** 1 Guangdong Provincial Key Laboratory of Marine Biology, Institute of Marine Sciences, Shantou University, 243 Daxue Road, Shantou 515063, China; 24yhlin@stu.edu.cn (Y.L.); 22ypwang@alumni.stu.edu.cn (Y.W.); 22qpan@alumni.stu.edu.cn (Q.P.); 19yyyuan@stu.edu.cn (Y.Y.); 2 College of Marine Sciences, South China Agricultural University, Guangzhou 510642, China; wenxbo@scau.edu.cn

**Keywords:** Chu’s croaker (*Nibea coibor*), cell culture, swim bladder cell line, TGF-β1, collagen regulation

## Abstract

Collagen is a major structural component of the extracellular matrix (ECM) and an important component contributing to the quality of fish maw. The collagen-rich swim bladder provides a suitable tissue source for investigating collagen regulation in fish. Therefore, systematic characterization of long-term cultured swim bladder-derived cells is needed to evaluate their utility as an in vitro model for investigating collagen regulation in fish. Here, we systematically characterized a previously established swim bladder-derived cell line from Chu’s croaker (*Nibea coibor*), designated NCSB, and examined its collagen-related responses to TGF-β1. NCSB cells exhibited fibroblast-like morphology and grew stably in DMEM/F12 medium with 15% FBS at 28 °C for over 40 passages. COI gene sequencing confirmed their species origin as *N. coibor*, while their fibroblast-like morphology and Vimentin expression were consistent with the mesenchymal-like phenotype. Transient transfection with pEGFP-N1 resulted in detectable GFP expression in NCSB cells. TGF-β1 treatment induced responses consistent with a myofibroblast-like phenotype, including increased α-SMA and collagen-related gene and protein expression, together with increased collagen-associated hydroxyproline in the culture supernatant and Sirius Red staining. Transcriptomic analysis further identified enrichment of the TGF-β signaling and ECM-receptor interaction pathways in TGF-β1-treated NCSB cells. Overall, the systematic characterization and functional evaluation of NCSB may support its utility as an in vitro model for studying collagen regulation in *N. coibor*.

## Simple Summary

Collagen is an important structural component contributing to the texture and quality of fish maw, an aquatic product derived from the fish swim bladder. The swim bladder, a collagen-rich organ, provides a relevant model for studying collagen regulation. Previous studies have used swim bladder-derived cells from Chu’s croaker (*Nibea coibor*) to investigate collagen-related processes, but these cells have not been systematically characterized for long-term culture. In this study, we systematically characterized NCSB, a previously established swim bladder-derived cell culture from *N. coibor*, and evaluated its long-term culture characteristics. NCSB cells grew stably and expressed collagen-related genes and proteins. Consistent with previous studies, TGF-β1 treatment increased collagen-related gene expression and collagen accumulation in NCSB cells. Transcriptomic analysis further showed identified enrichment of the TGF-β signaling and ECM-receptor interaction pathways in response to TGF-β1 treatment. NCSB cells may therefore provide a useful laboratory model for studying collagen regulation in fish.

## 1. Introduction

Collagen is the most abundant structural protein in animals and serves as the primary constituent of the extracellular matrix (ECM), playing an essential role in maintaining tissue mechanical strength, elasticity and structural integrity [1]. Owing to its excellent biocompatibility and low immunogenicity, collagen is widely applied in the food industry and biomedical materials [2,3,4]. Marine-derived collagen closely resembles mammalian collagen in amino acid composition and physicochemical properties while posing lower disease-transmission risk and fewer religious or cultural restrictions, making it an important alternative source [5,6]. In aquatic animals, collagen significantly influences key quality attributes such as texture, elasticity and nutritional value [7,8]. Aquaculture conditions and nutritional interventions can influence collagen deposition in fish, but such in vivo studies are often time-consuming and subject to environmental variation and stress [9,10,11]. Thus, an in vitro cell model can complement in vivo studies by providing a controlled experimental system for investigating cellular responses associated with collagen regulation.

Elucidating the cellular and molecular mechanisms underlying collagen regulation and deposition is essential for the precise regulation of aquatic product quality. The homeostasis of the ECM largely relies on a dynamic balance between collagen synthesis and degradation [12,13]. In mammals, transforming growth factor-β1 (TGF-β1) is widely recognized as a key regulator of collagen production, playing a central role in collagen synthesis, ECM deposition and tissue remodeling [14,15,16]. As an important member of the TGF-β superfamily, TGF-β1 exerts diverse biological functions through both canonical Smad-dependent and non-Smad signaling pathways [17]. TGF-β1 upregulates downstream effectors such as connective tissue growth factor (CTGF), α-smooth muscle actin (α-SMA) and integrins, further amplifying its pro-collagenic effects [18]. Beyond directly promoting collagen synthesis, TGF-β1 also modulates the expression and activity of matrix metalloproteinases (MMPs) and their endogenous inhibitors (TIMPs), thereby influencing the balance between ECM degradation and deposition [19,20]. Previous studies have also investigated TGF-β-related collagen regulation in fish, including TGF-β/Smad signaling in swim bladder-derived cells from *N. coibor* [21,22]. These findings provide a basis for the further investigation of collagen regulation in fish cell models.

Fish cell cultures, owing to their experimental accessibility and controllability, have become useful in vitro models for studying cellular physiology, molecular regulation and tissue-specific biological processes [23,24]. To date, researchers have established numerous fish cell lines from various tissues, including gill [25], heart [26], liver [27], muscle [28] and skin [29], widely applied in virological, toxicological, immunological and molecular biology research. In contrast, fish cell lines derived from collagen-rich connective tissues remain relatively limited. The swim bladder, a gas-filled organ found in certain fish species that primarily regulates buoyancy, is characterized by abundant collagen and a highly organized ECM structure, making it a useful tissue source for studying collagen synthesis and deposition [11]. However, swim bladder-derived cell lines have been reported in only a few fish species [11,30,31,32,33,34,35,36] (Table S1). Most previously reported swim bladder-derived cell lines have been used for viral susceptibility studies, whereas their application in collagen-related research remains limited. Notably, swim bladders from several sciaenid species have been reported to be rich in collagen [37,38]. Nevertheless, the long-term culture properties and biological characteristics of sciaenid swim bladder-derived cells remain insufficiently documented.

Among sciaenid fish, *Nibea coibor* is a marine species widely distributed in the South China Sea, the East China Sea, and adjacent Indo-Pacific waters [39]. Characterized by its large swim bladder, which is commercially valuable as fish maw, this species is a major source of this highly valued aquatic product [11,40]. Dried fish maw has been consumed in Asia for centuries and remains particularly popular in southern China [41]. As a major component of fish maw, collagen is closely associated with its quality and commercial value. Consequently, investigating collagen regulation in the swim bladder may contribute to a better understanding of the biological factors associated with fish maw quality. In our previous studies, we used NCSB cells to investigate collagen-related regulation [11,21]. In particular, a recent study showed that TGF-β1 upregulated Smad3 expression in swim bladder-derived cells and that Smad3 enhanced the promoter activities of col1a1 and col1a2 [22]. Building on these findings, the present study aimed to systematically characterize the biological features and long-term culture properties of the previously established NCSB cell line and evaluate its collagen-related responses to TGF-β1. Transcriptomic analysis was further performed to explore the broader gene expression changes associated with the TGF-β1 response in NCSB cells.

## 2. Materials and Methods

### 2.1. Ethics Statement

All animal study protocols were approved by the Institutional Animal Care and Use Ethics Committee of Shantou University (Approval No. 2022132, 20 July 2022). All efforts were made to minimize the suffering of the animals. All experiments were performed in accordance with relevant guidelines and regulations.

### 2.2. Primary Culture and Subculture

The NCSB cell line was previously established from the swim bladder of *N. coibor* by tissue explant culture, following the primary culture protocol described in our previous study [11]. The present study further characterized this existing cell resource and its long-term culture properties. The original primary culture was derived from the swim bladder of a single healthy, approximately 6-month-old *N. coibor* (body weight approximately 50 g) collected from Nan’ao Marine Biology Station (Shantou University, Shantou, China). A single independent primary culture was established from this individual fish, and no tissues from other individuals were pooled during cell establishment. Prior to the experiment, the fish was acclimated for 24 h in a laboratory tank containing penicillin and streptomycin. The fish was then euthanized with eugenol (Sinopharm Chemical Reagent Co., Ltd., Shanghai, China) and surface-disinfected with 70% ethanol for 2 min. The disinfected fish was transferred to a clean bench and disinfected again with 75% ethanol-soaked cotton balls. The abdominal cavity was opened with sterile scissors, the viscera were removed, and the swim bladder was carefully dissected out with sterile forceps and freed of adherent fat and blood vessels. The isolated swim bladder tissue was washed three times with phosphate buffer solution (PBS, Biosharp, Hefei, China) containing 1% penicillin–streptomycin. Using sterile surgical scissors, the tissue was mechanically minced into fragments of about 1 mm^3^ in a Petri dish containing DMEM/F12 (1:1; Gibco, Grand Island, NY, USA) supplemented with 5% fetal bovine serum (FBS, Gibco, Grand Island, NY, USA). The tissue fragments were washed twice with serum-free DMEM/F12, pelleted by centrifugation at 800 rpm for 5 min, and seeded into T25 flasks pre-coated with 0.1% gelatin (Sigma-Aldrich, St. Louis, MO, USA). After a 6-h attachment period, 1 mL of complete DMEM/F12 medium was added gently to avoid dislodging the tissue fragments. The complete medium consisted of 20% FBS, recombinant growth factors (20 ng/mL bFGF, 20 ng/mL EGF, and 40 ng/mL IGF-I; GenScript, Nanjing, China), and antibiotics. An additional 3 mL of complete medium was added after 24 h. Cultures were maintained at 28 °C, with 2 mL of medium replaced every 3 days. When cells reached approximately 90% confluence, they were subcultured using 0.25% trypsin-EDTA (Transgen, Beijing, China) at a split ratio of 1:2. After passage 10, the FBS concentration was reduced to 15%, and the medium was supplemented only with penicillin and streptomycin.

### 2.3. Cryopreservation and Resuscitation

For cryopreservation, NCSB cells were pelleted by centrifugation, and resuspended in 1 mL of freezing medium consisting of 700 μL DMEM/F12, 100 μL DMSO, and 200 μL FBS. Cell suspensions in sterile cryovials were gradually frozen in a controlled-rate freezing container before storage in liquid nitrogen. To recover frozen cells, cryovials were quickly warmed in a 37 °C water bath, washed to remove freezing medium, and resuspended in DMEM/F12 with 15% FBS. Cell viability was determined by trypan blue exclusion using a hemocytometer and calculated as the percentage of unstained cells among the total counted cells. Cells not used for counting were cultured in T25 flasks at 28 °C.

### 2.4. Optimization of Cell Medium

Growth characteristics of the established cell line were assessed at 28 °C across different serum concentrations and culture media. To optimize culture conditions, cells were grown in DMEM/F12 containing 5%, 10%, 15%, or 20% FBS and monitored for 6 days. Additionally, 4-day growth comparisons were performed in Dulbecco’s modified Eagle’s medium (DMEM, Gibco, Grand Island, NY, USA), DMEM/F12, and Leibovitz’s L15 (L15, Gibco, Grand Island, NY, USA), all supplemented with 15% FBS. For all assays, cells were seeded in 96-well plates at approximately 2 × 10^3^ cells per well, with six replicate wells per treatment group at each time point. Separate wells were designated for each measurement time point. Cell growth was assessed using the Cell Counting Kit-8 (CCK-8, Transgen, Beijing, China). CCK-8 reagent was added to each well at 10% of the final volume. After incubation in the dark for 2 h, absorbance at 450 nm was measured using a microplate reader. Cell-free blank wells were included, and background absorbance was subtracted from the sample readings. The mean absorbance value of the six replicate wells was used to generate the growth curve.

### 2.5. Species Identification of NCSB Cells

Genomic DNA of the NCSB cell line was extracted using the FastPure Cell/Tissue DNA Mini Kit (Vazyme, Nanjing, China) to confirm its species origin. Cells were harvested by centrifugation, resuspended in 220 μL PBS, and digested with 10 μL RNase A and 20 μL Proteinase K at room temperature for 15 min, followed by lysis in Buffer GB at 65 °C for 30 min. After ethanol addition, the lysate was loaded onto a gDNA column, washed with Washing Buffers A and B, and eluted with 30 μL of pre-warmed elution buffer. Purified DNA was stored at −20 °C until use. Polymerase chain reaction (PCR) was performed to amplify the mitochondrial cytochrome c oxidase subunit I (COI) gene fragment using primers VF1-t1 (5′-TCTCAACCAACCACAAAGACATTGG-3′) and VR1-t1 (5′-TAGACTTCTGGGTGGCCAAAGAATCA-3′) [42]. PCR was carried out in a 50 μL reaction volume consisting of 2 μL of each primer, 1 μL DNA template, 25 μL 2× Phanta Max premix (Takara Bio Inc., Dalian, China), and nuclease-free water to make up the volume. The PCR program comprised an initial denaturation at 94 °C for 3 min, followed by 35 cycles of denaturation (94 °C, 30 s), annealing (52 °C, 50 s), and extension (72 °C, 1 min), with a final extension step at 72 °C for 8 min. The products were sent to Sangon, Inc. (Shanghai, China) for sequencing. The obtained sequences were then aligned with reference sequences from the NCBI database.

### 2.6. Mycoplasma Screening

Mycoplasma contamination in NCSB cultures was detected using a one-step mycoplasma detection kit (Servicebio Technology Co., Ltd., Wuhan, China). Supernatants were collected from cells cultured in six-well plates for 4 days. Each 25 μL reaction contained 23 μL Mycored Buffer, 1 μL Mycored Enzyme, and 1 μL of sample, and was overlaid with 20 μL paraffin oil, with the sample added beneath the oil layer. Alongside the test samples, a tube receiving 1 μL positive control served as the positive control, and a tube without sample served as the negative control. A yellow reaction solution was considered negative, and a red solution was considered positive.

### 2.7. DNA Transfection

The ability of NCSB cells to express an exogenous reporter gene was evaluated by transient transfection with the plasmid pEGFP-N1. Cells were seeded in 12-well plates and transfected at 60% confluence using Hieff Trans^®^ Universal Transfection Reagent (YEASEN, Shanghai, China) according to the manufacturer’s instructions. Briefly, 1 μg EGFP-N1 plasmid was diluted in Opti-MEM medium and mixed with the transfection reagent in Opti-MEM at the recommended ratio. The mixture was incubated at room temperature for 5 min and then added to the cells. After 8 h, the medium was replaced with fresh complete medium, and the cells were further incubated at 28 °C for 48 h. Green fluorescent protein (GFP) expression was visualized using a fluorescence microscope (ZEISS, Oberkochen, Germany) to assess exogenous reporter gene expression.

### 2.8. Immunofluorescence Staining

NCSB cells were seeded on glass coverslips placed in 12-well plates and cultured for 24 h. Cells were fixed with 4% paraformaldehyde Fix Solution (PFA) for 20 min at room temperature, then washed again with PBS. Following fixation, cells were permeabilized with PBS containing 0.5% Triton X-100 (PBST) and blocked using cell blocking solution (Beyotime, Shanghai, China) for 90 min at room temperature. After washing with PBS, NCSB cells were incubated with primary anti-Vimentin antibody (1:150, A11952, ABclonal, Wuhan, China) overnight at 4 °C. Subsequently, the cells were incubated with FITC-conjugated Goat anti-Rabbit IgG (H + L) (1:250, AS011, ABclonal, Wuhan, China) for 1 h at room temperature, washed with 0.1% PBST, and counterstained with DAPI (Beyotime, Shanghai, China). After three additional PBST washes, coverslips were mounted onto glass slides and fluorescent signals were captured using a confocal laser scanning microscope (ZEISS, Oberkochen, Germany).

### 2.9. TGF-β1 Treatment

To evaluate the response of NCSB cells to TGF-β1, cells were seeded into 96-well plates at a density of approximately 2 × 10^3^ cells per well. After the cells had adhered, the culture medium was replaced with fresh complete medium containing various concentrations of TGF-β1 (0, 5, 10, 20, and 50 ng/mL; GenScript, Nanjing, China), and the cells were incubated at 28 °C. Cell growth was assessed over 5 days using the CCK-8 assay as described above. Additionally, under 10 ng/mL TGF-β1 treatment conditions, the effects of different incubation times (24 h and 48 h) on collagen-related gene expression were also analyzed. Furthermore, following 48 h of stimulation with 10 ng/mL TGF-β1, cells were collected for RNA extraction, collagen content determination, Western blot analysis, picrosirius red staining and transcriptome sequencing.

### 2.10. Quantitative Real-Time PCR (qRT-PCR)

NCSB cells were subjected to total RNA extraction with TRIzol reagent (Invitrogen, Carlsbad, CA, USA) according to the manufacturer’s instructions, and cDNA was subsequently synthesized from 1 μg of total RNA using HiScript II Q Select RT SuperMix (Vazyme, Nanjing, China). Then, using ChamQ SYBR qPCR Master Mix (Vazyme, Nanjing, China), qRT-PCR was carried out on a LightCycler 480 II system (Roche, Mannheim, Germany). Relative gene expression was determined by normalizing to 18S rRNA and applying the 2^−ΔΔCt^ method. Six biological replicates were used for each group. Primer details are shown in Table 1.

### 2.11. Collagen Content Determination

As a collagen-specific amino acid, hydroxyproline (Hyp) can be used as an indicator of collagen content [43]. In this study, total collagen content in culture supernatants collected after 48 h of TGF-β1 treatment was determined using a Hydroxyproline Assay Kit (Nanjing Jiancheng Bioengineering Institute, Nanjing, China). Briefly, 0.5 mL of supernatant was mixed with 0.05 mL of digestion solution and incubated at 37 °C for 3 h. Following digestion, 0.5 mL of Reagent I was added and the mixture was kept at room temperature for 10 min. Reagent II (0.5 mL) was then added, and after a further 5 min, 1.0 mL of Reagent III was added. After incubation at 60 °C for 15 min, the mixture was cooled and centrifuged at 3500 rpm for 10 min. After measuring Hyp concentration at 550 nm via a microplate reader, collagen content was measured based on the instructions provided with the kit.

### 2.12. Western Blot Analysis

NCSB cells were lysed with RIPA buffer (Beyotime, Shanghai, China) supplemented with protease inhibitors, and the resulting protein lysates were quantified using a BCA Protein Assay Kit (Beyotime, Shanghai, China). Specifically, the working reagent was prepared by mixing reagent A and reagent B from the BCA kit at 50:1. Each sample (20 μL) was mixed with 200 μL of working reagent and incubated at 37 °C for 30 min, and the absorbance was measured at 562 nm. Protein concentrations were calculated from a standard curve. Samples of equal quantity were supplemented with 5× SDS–PAGE loading buffer (Beyotime, Shanghai, China), boiled at 100 °C for 10 min, followed by separation on an 8% SDS–PAGE gel. Each sample was loaded with 20 μg of protein. The resolved proteins were electrophoretically transferred onto PVDF membranes (Vazyme, Nanjing, China).

The membrane was blocked with TBST (Biosharp, Hefei, China) containing 5% skim milk for 90 min at room temperature. The membrane was then washed three times with TBST for 5 min each. Subsequently, the membrane was incubated with primary antibodies for 2 h at room temperature. The primary antibodies including anti-tubulin (1:3000, AT819, Beyotime, Shanghai, China), anti-collagen type I (1:1000, the antibody was custom-synthesized by GenScript according to the sequence provided by our laboratory), anti-α-smooth muscle actin (1:1000, abs130621, Absin, Shanghai, China), and anti-Vimentin (1:1000, T55134, Abmart, Shanghai, China). The membrane was then washed three times with TBST for 5 min each. The washed membranes were subjected to 1 h incubation with HRP-conjugated secondary antibodies. Goat anti-rabbit IgG (1:7500, AS014, ABclonal, Wuhan, China) and goat anti-mouse IgG (1:7500, AS003, ABclonal, Wuhan, China) were used as appropriate. After incubation, the membrane was washed four times with TBST for 5 min each. Signals were detected with an ECL kit (Meilunbio, Dalian, China) and quantified by densitometry using ImageJ software (v1.54g). Relative expression levels were normalized to the grayscale intensity of the loading control tubulin by calculating the ratio of target protein to tubulin.

### 2.13. Picrosirius Red Staining

Collagen visualization was performed using Sirius Red staining. As Liu described previously [44], NCSB cells were fixed with a fixative consisting of absolute ethanol and glacial acetic acid at a ratio of 95:5 (*v*/*v*) for 20 min. After a PBS wash, cells were incubated with 0.1% Sirius Red (Biosharp, Hefei, China) for 1 h at room temperature on an orbital shaker, followed by a rinse with 0.5% acetic acid to remove unbound dye. The cells were then dehydrated through a graded ethanol series, cleared, and mounted on slides, and images were captured under bright-field illumination using a Zeiss microscope (Zeiss, Oberkochen, Germany).

### 2.14. Transcriptome Analysis and Validation

Total RNA was isolated from the control and TGF-β1-treated (10 ng/mL) NCSB cells with TRIzol reagent (Invitrogen, Carlsbad, CA, USA) according to the manufacturer’s instructions. RNA structural integrity was confirmed through Bioanalyzer analysis 2100 (Agilent Technologies, Santa Clara, CA, USA). For each treatment group, six independent biological samples were collected. Equal amounts of total RNA from three biological samples were pooled to generate one RNA-Seq library, resulting in two pooled libraries per treatment group. The resulting cDNA libraries were sequenced on an Illumina NovaSeq platform (Illumina, San Diego, CA, USA). As a reference genome for *N. coibor* was not available at the time of the analysis, clean reads obtained after quality filtering were subjected to de novo transcriptome assembly using Trinity (v2.5.1) [45] to generate unigenes. Gene functional annotation was performed through alignment with multiple databases, including Nr, Pfam, Swiss-Prot, KOG/COG, EggNOG, and GO. Clean reads were mapped to the assembled unigene sequences using Bowtie2 (v2.3.5.1), and gene expression levels were estimated using RSEM (v1.2.19) [46]. Fragments per kilobase of transcript per million mapped reads (FPKM) values were used to describe gene expression abundance.

Differential expression analysis was performed using DESeq2 (v1.6.3) based on RSEM-derived count data [47]. Genes with |log_2_FC| ≥ 0.585 and a false discovery rate (FDR) < 0.05 were considered differentially expressed. The identified DEGs were subjected to Gene Ontology (GO) functional classification to characterize their distribution across biological process, cellular component, and molecular function categories. Thirty GO terms were selected for visualization. KEGG pathway enrichment analysis (http://www.genome.ad.jp/kegg/ accessed on 11 May 2025) was performed using a hypergeometric test, with q-values used to account for multiple testing. Pathways with a q-value < 0.05 were considered enriched.

qRT-PCR was performed to further assess the consistency of selected RNA-Seq findings, focusing on five genes related to the TGF-β signaling pathway: Bone Morphogenetic Protein Receptor 1 (*BMPRI*), Inhibitor of DNA Binding (*Id*), Mothers Against Decapentaplegic Homolog 1 (*Smad1*), Transforming Growth Factor Beta (*TGFβ*), and Thrombospondin 1 (*THBS1*). Five independent biological replicates were used for each group. Primer details are shown in Table 1.

### 2.15. Statistical Analysis

GraphPad Prism version 10.1.2 and SPSS version 26 (SPSS Inc., Chicago, IL, USA) were used for statistical analyses and graphical visualization. Data were assessed for normality using the Shapiro–Wilk test and homogeneity of variance using Levene’s test. Differences between two groups were analyzed using Student’s *t*-test, while multiple-group comparisons were performed using one-way ANOVA followed by Tukey’s post hoc test. CCK-8 growth curves were presented descriptively without statistical significance testing. Data are presented as mean ± SEM, and sample sizes (*n*) are indicated in the corresponding figure legends. *p* < 0.05 was considered statistically significant.

## 3. Results

### 3.1. Establishment and Optimization of a Swim Bladder Cell from Nibea coibor

**Figure 1:**
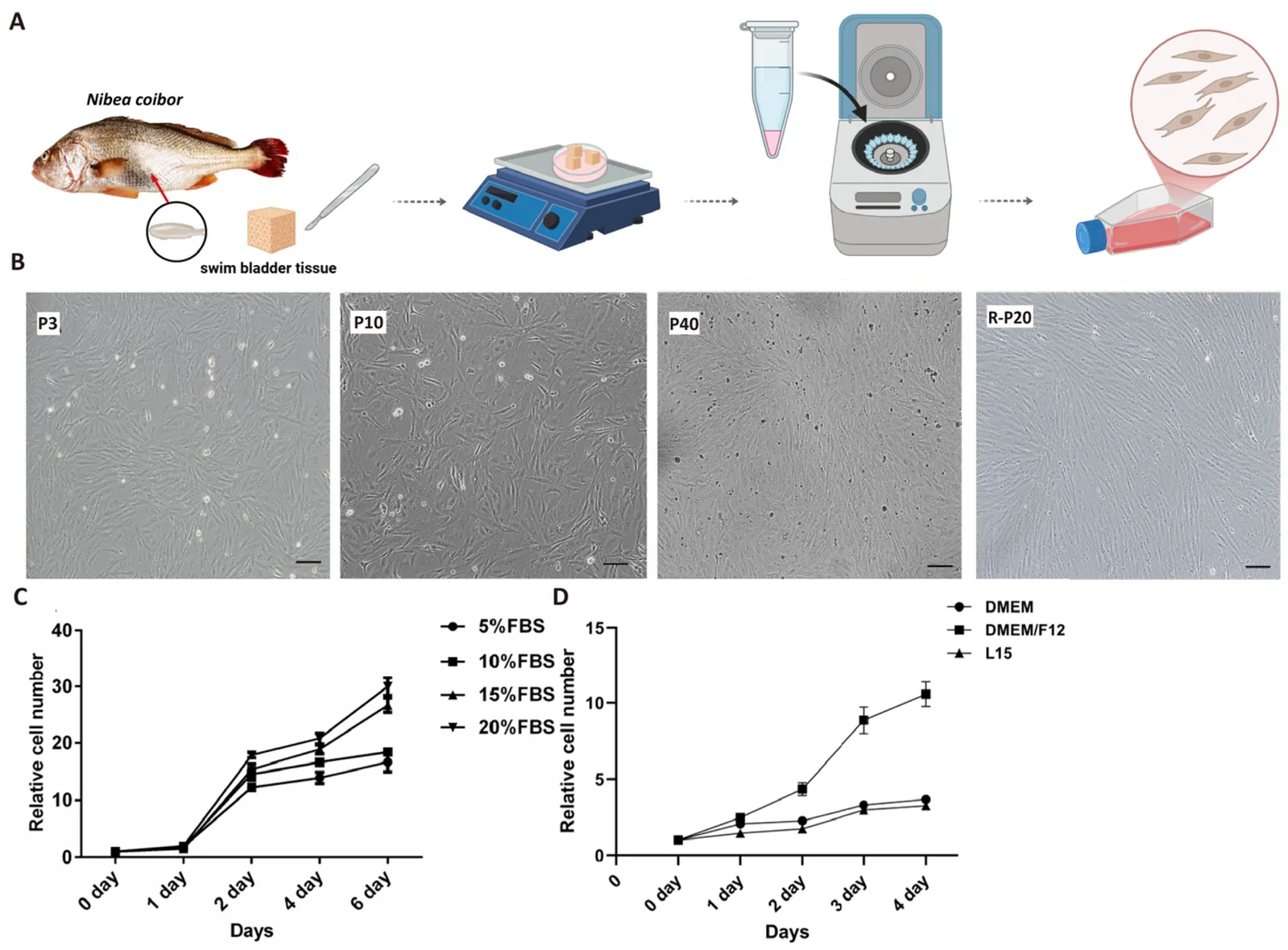
Establishment and culture characterization of the NCSB cell line. (**A**) Flowchart for the primary culture of *N. coibor* swim bladder tissue. (**B**) Representative micrographs of NCSB cells at various passages (P3–P40) and at passage 20 after cryopreservation and recovery. Scale bar, 100 μm. (**C**) Growth curves of NCSB cells cultured in DMEM/F12 supplemented with 5%, 10%, 15%, or 20% FBS. (**D**) Growth curves of NCSB cells cultured in DMEM, DMEM/F12, or L15 supplemented with 15% FBS.

The workflow for isolating swim bladder cells from the swim bladder tissue of *N. coibor* is briefly illustrated in Figure 1A. Primary cells migrated from the swim bladder tissue explants within 3 days of plating. The migrated cells exhibited a spindle-shaped morphology and grew relatively rapidly, reaching approximately 90% confluence within 14 days at 28 °C. Early-stage cells displayed heterogeneous morphology but were generally spindle-shaped (Figure 1B). By passage 10, the majority of cells exhibited a fibroblast-like morphology. Cells were subcultured every 3–5 days at a split ratio of 1:2 or 1:3 during subsequent passages. To date, cells have been successfully subcultured over 40 passages, with cell morphology gradually becoming uniformly fibroblast-like. The cells were tested for mycoplasma contamination, and the results were negative (Figure S1). After six months of cryopreservation, NCSB cells exhibited a post-thaw viability of 86%, with no notable changes in cellular morphology.

The effects of different FBS concentrations on NCSB cell growth are shown in Figure 1C. NCSB cell growth generally increased with increasing FBS concentration within the tested range of 5–20%. Between days 4–6, cell growth tended to plateau at FBS concentrations of 5% and 10%, whereas cells cultured with 15% and 20% FBS maintained higher proliferative activity. By day 6, cell growth reached the highest level in the 20% FBS group. Considering both the overall growth pattern and FBS consumption, 15% FBS was selected for the subsequent culture of NCSB cells.

The effects of various culture media on NCSB cell growth are shown in Figure 1D. In all three media supplemented with 15% FBS, NCSB cells grew progressively over time. However, cell growth differed among the three media. NCSB cells exhibited higher proliferative activity in DMEM/F12 than in DMEM or L15. By day 4 of culture, the DMEM/F12 group showed the highest level of cell growth, whereas cells cultured in DMEM or L15 showed lower proliferative activity. These results indicate that DMEM/F12 was more favorable for NCSB cell growth under the tested conditions.

### 3.2. Molecular and Biological Characterization of the NCSB Cell

**Figure 2:**
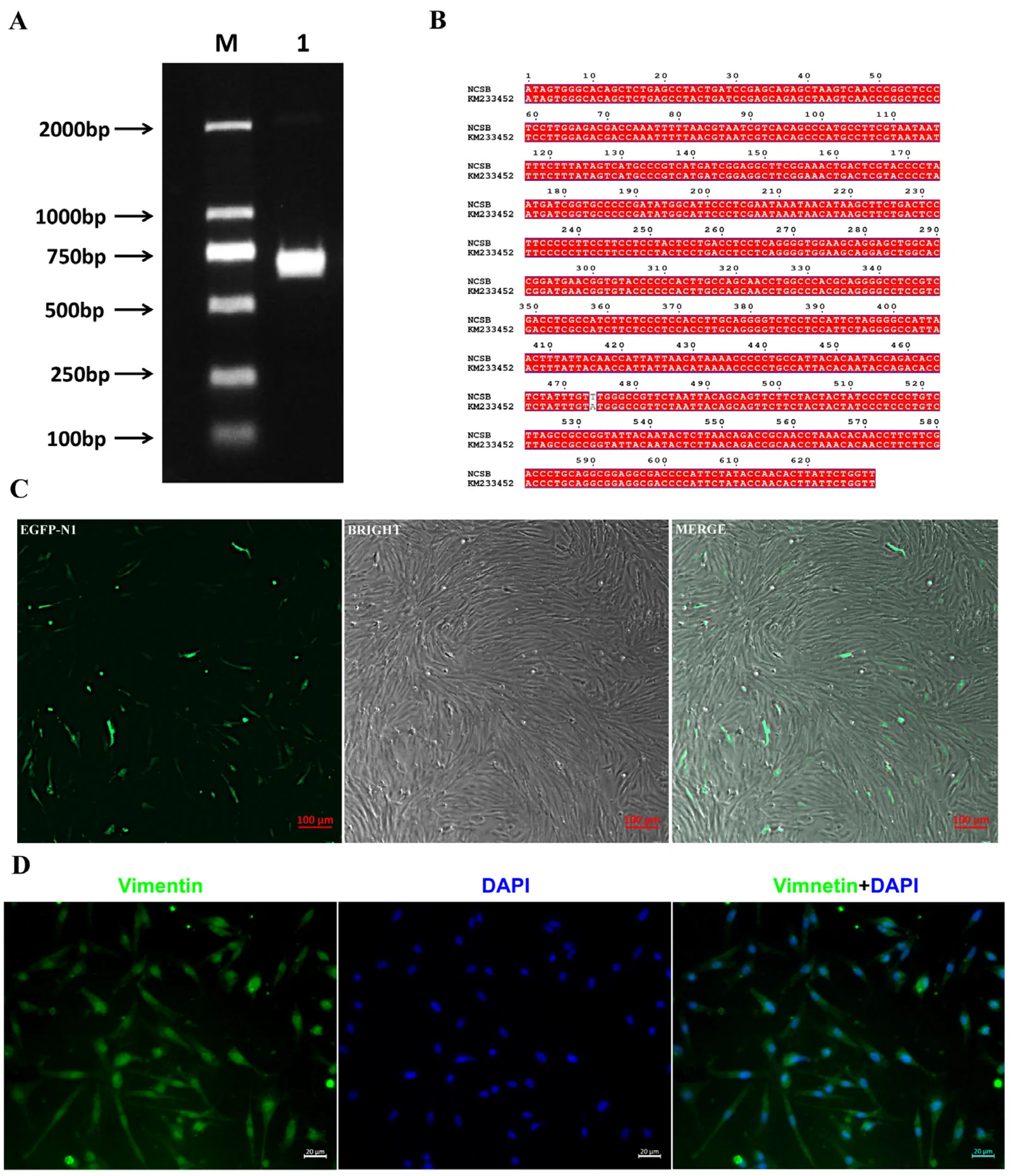
Molecular and cellular characterization of NCSB cells. (**A**) PCR amplification of the COI gene fragment. Lane M: DNA molecular weight marker (2000 bp); Lane 1: PCR product of the COI gene amplified from NCSB cells. (**B**) Nucleotide sequence of the COI gene fragment amplified in (**A**). (**C**) Fluorescence microscope photographs of NCSB cells transfected with the pEGFP-N1 plasmid. Scale bar, 100 μm. (**D**) Expression of Vimentin in NCSB cells. Immunofluorescence staining of Vimentin (green) and nucleus (DAPI, blue) in NCSB cells. Scale bar, 20 μm.

Successful amplification of a 658-bp COI gene fragment from NCSB cell DNA was achieved (Figure 2A). Sequence alignment revealed over 99% identity between the obtained COI sequence and the reference sequence of *N. coibor* (GenBank: KM233452.1; Figure 2B), supporting the species origin of NCSB cells.

The expression of an exogenous reporter gene in NCSB cells was examined by transfection with the pEGFP-N1 plasmid via lipofection. GFP-positive cells were observed 48 h post-transfection (Figure 2C), indicating successful expression of the exogenous reporter gene in NCSB cells.

**Figure 3:**
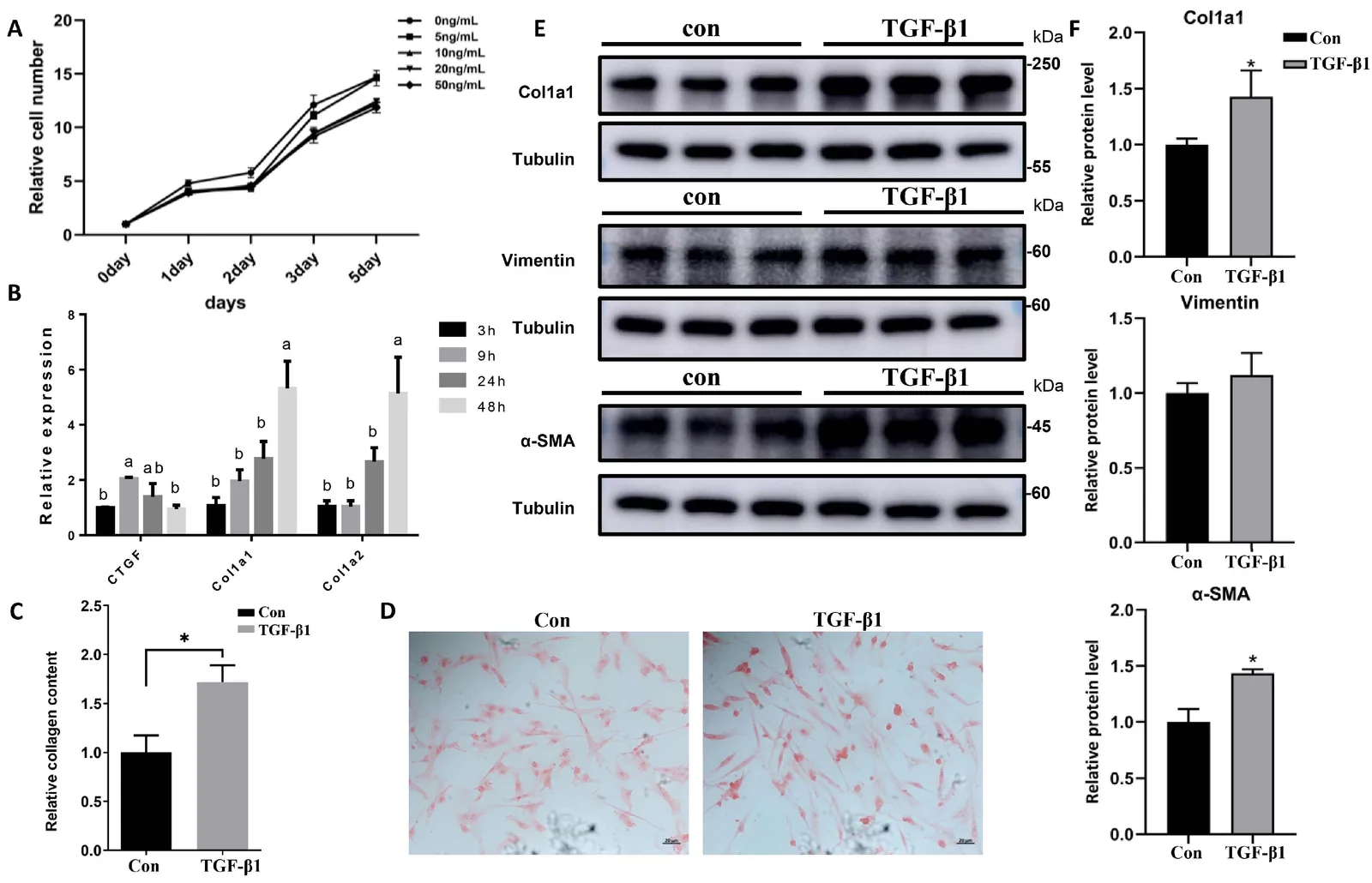
Effects of TGF-β1 on collagen-related expression and accumulation in NCSB cells. (**A**) Cell proliferation after treatment with different concentrations of TGF-β1 for 5 days. (**B**) Relative expression levels of collagen synthesis-related genes after treatment with 10 ng/mL TGF-β1 for different durations (*n* = 6). (**C**) Relative collagen content after treatment with 10 ng/mL TGF-β1 for 48 h (*n* = 3). (**D**) Sirius Red staining of swim bladder cells treated with TGF-β1 (10 ng/mL). Scale bars, 20 μm. (**E**) Expression of fibrosis-related and Col1a1 proteins after treatment with 10 ng/mL TGF-β1 for 48 h (*n* = 3). (**F**) Quantification of protein expression normalized to tubulin (*n* = 3). Bars marked with * or different letters indicate significant differences (*p* < 0.05).

Vimentin expression was detected in NCSB cells by immunofluorescence staining and Western blotting (Figure 2D and Figure 3E). Together with their fibroblast-like morphology, these findings were consistent with mesenchymal-like characteristics.

### 3.3. TGF-β1 Increases Collagen-Related Expression and Accumulation in NCSB Cells

Using the previously established NCSB cell culture, we investigated its response to TGF-β1 to evaluate its potential for studying collagen regulation.

The effects of different TGF-β1 concentrations on cell proliferation were evaluated first (Figure 3A). The results showed that cells in all treatment groups exhibited growth trends over the 5-day culture period, indicating overall good cell viability. Compared with the control group (0 ng/mL), TGF-β1-treated cells showed slightly lower relative cell viability at each time point. This difference was more evident at higher concentrations (10–50 ng/mL) on days 3–5.

Compared with the control group (0 ng/mL), treatment with 10 ng/mL TGF-β1 resulted in a slight reduction in cell number during the first 48 h, while NCSB cells continued to proliferate. Considering that 10 ng/mL maintained acceptable cell growth while being within the concentration range previously reported to induce fibroblast activation, this concentration was selected as a representative concentration for subsequent experiments [48,49]. Collagen-related gene expression was assessed at 24 and 48 h to examine the response within the selected treatment period, and 48 h was used for subsequent molecular and collagen-related analyses. After treatment of the NCSB cells with TGF-β1 (10 ng/mL), the expression levels of collagen-related genes at different time points were analyzed by qRT-PCR (Figure 3B). The results indicated that the expression level of *CTGF* exhibited a dynamic pattern of initial increase followed by decrease, while the expression of genes encoding type I collagen (*Col1a1* and *Col1a2*) continued to increase with prolonged treatment time. Subsequently, collagen content in the culture supernatant was estimated based on hydroxyproline measurements, and collagen deposition was visualized by Sirius Red staining. The collagen-associated hydroxyproline in the culture supernatant was significantly increased after 48 h of TGF-β1 treatment compared with the control group (Figure 3C). More intense Sirius Red staining was also observed following TGF-β1 treatment (Figure 3D). Western blot analysis further showed that TGF-β1 treatment significantly increased α-SMA and COL1A1 protein expression, whereas Vimentin expression remained unchanged (Figure 3E,F). Together, these findings indicate that TGF-β1 treatment increased collagen-related gene and protein expression and was associated with a greater accumulation of collagen-related material in NCSB cultures.

### 3.4. Transcriptomic Responses of NCSB Cells to TGF-β1 Treatment

**Figure 4:**
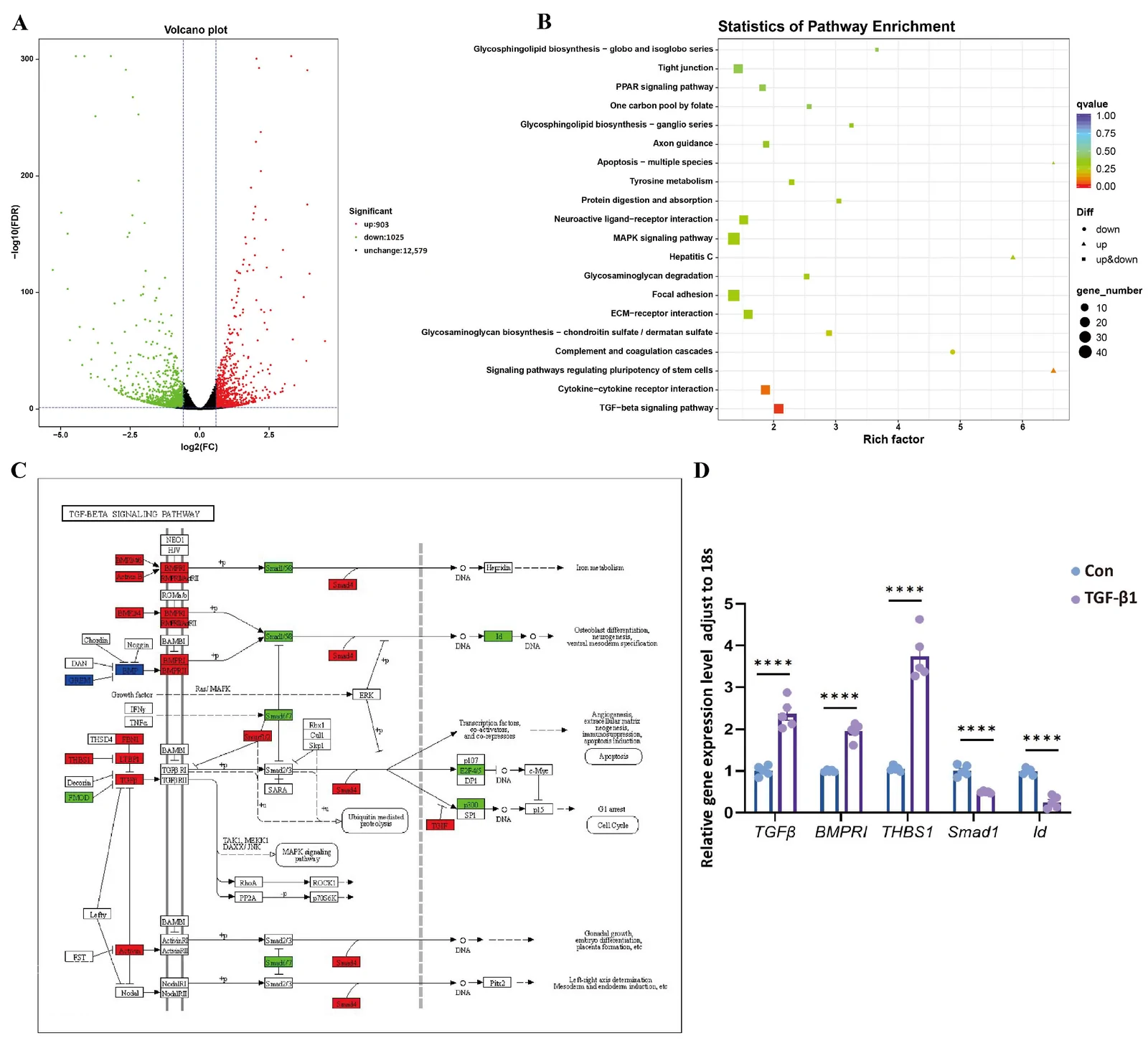
Transcriptomic analysis of NCSB cells following TGF-β1 treatment. (**A**) Volcano plot of DEGs showing upregulated and downregulated genes in TGF-β1-treated cells compared to controls. (**B**) KEGG pathway enrichment statistics of DEGs. (**C**) DEGs enriched in the TGF-beta signaling pathway. Red, green, and blue denote upregulated, downregulated, and bidirectionally regulated genes, respectively. (**D**) Validation of selected DEGs by qRT-PCR (*n* = 5). Data are shown as mean ± SEM; ****, *p* < 0.0001.

RNA-Seq analysis was performed to examine the transcriptional responses of NCSB cells to TGF-β1 treatment. Based on the predefined differential expression criteria (|log2FC| ≥ 0.585 and FDR < 0.05), 1928 DEGs were identified between the TGF-β1-treated and control groups, including 903 upregulated and 1025 downregulated genes (Figure 4A).

Gene Ontology (GO) functional classification was performed to characterize the distribution of DEGs across three major categories: biological process (BP), cellular component (CC), and molecular function (MF). A total of 1338 DEGs were annotated to 53 GO terms across these categories, of which 30 are presented in Figure S2. In the BP category, cellular process, single-organism process, and biological regulation contained the largest numbers of DEGs. In the CC category, the DEGs were primarily assigned to cell, cell part, and membrane. In the MF category, binding and catalytic activity were the most represented terms.

KEGG pathway enrichment analysis identified 20 enriched pathways, including the “TGF-beta signaling pathway”, “Cytokine–cytokine receptor interaction”, “Signaling pathways regulating pluripotency of stem cells”, and “ECM-receptor interaction” (Figure 4B).

To further assess the consistency of the selected RNA-Seq findings, five DEGs related to TGF-β signaling, including *TGFβ*, *BMPRI*, *THBS1*, *Smad1*, and *Id*, were selected for qRT-PCR analysis. The expression patterns of these five genes determined by qRT-PCR were consistent with those observed in the RNA-Seq analysis (Figure 4D). Together, these findings suggest that TGF-β-related signaling may be involved in the response of NCSB cells to TGF-β1 treatment, supporting the potential utility of this culture as an in vitro model for investigating collagen regulation in fish.

## 4. Discussion

Since Wolf and Quimby established RTG-2, the first continuous fish cell line derived from the gonad of *Salmo gairdneri*, in 1962, numerous additional fish cell lines have been developed [50]. Previous studies have used *N. coibor* swim bladder-derived cells to investigate collagen-related responses [11,21,22]. The NCSB culture characterized in the present study represents a continuation of the same primary culture used in our previous investigations. Building on these previous studies, we further characterized the culture conditions, long-term culture characteristics, species origin, exogenous gene expression capability, and responsiveness to TGF-β1 of this swim bladder-derived culture. These findings indicate that NCSB may provide a useful in vitro model for investigating collagen-related regulation in fish.

### 4.1. Establishment and Characterization of the NCSB Culture

Primary animal cells are mainly isolated using two methods, tissue explant culture and enzymatic dissociation. The former is simpler to operate, has a higher success rate, and avoids damage to primary cells caused by trypsin digestion [51]. The NCSB culture used in the present study was originally established using the tissue explant method, with primary cells reaching approximately 90% confluence after 14 days. The culture has been maintained for more than 40 passages and continues to exhibit proliferative capacity and predominantly fibroblast-like morphology. Although this long-term passaging demonstrates the sustained growth capacity of NCSB cells under the present culture conditions, their immortalization has not been established. Morphological observation revealed that NCSB cells were heterogeneous during early passages, but predominantly fibroblast-like cells emerged after passage 10. Similar morphological changes have been observed in swim bladder cell lines from *C. striatus* [34] and *E. akaara* [35]. These observations suggest that the morphological composition of swim bladder-derived cultures may change during serial passaging, although cell lineage cannot be established from morphology alone.

In cell culture, appropriate medium components promote rapid cell growth, and cells from different sources have varying nutritional requirements. NCSB cell growth increased with increasing FBS concentration within the tested range, with the 20% FBS group showing the highest level of cell growth by day 6, similar to findings in gill cells of *Megalobrama amblycephala* [52] and skin cells of *Cynoglossus semilaevis* [29]. L15, DMEM, and DMEM/F12 are commonly used media for fish cell culture. Here, we found that NCSB cells grew better in DMEM/F12 medium compared to L15 and DMEM media.

The mitochondrial COI gene is commonly used as a DNA barcode for identifying the species origin of fish cell cultures [53,54]. The COI sequence obtained from NCSB cells showed high similarity to the reference sequence of *N. coibor* in the NCBI database, supporting the identification of *N. coibor* as the species of origin. Transfection potential is crucial for cell lines used in gene function studies in vitro. A transfection efficiency of approximately 40% has been reported for the zebrafish cell line ZBE3 [55]. In the present study, pEGFP-N1 was successfully expressed in NCSB cells following lipofection. Although transfection efficiency was not quantitatively assessed, it can be influenced by factors such as promoters, lipid complexes, and DNA incubation time [56,57]. Further quantitative evaluation and optimization of transfection conditions would help characterize the transfection performance of NCSB cells for future gene function studies. Additionally, as a major component of the intermediate filament family of the cytoskeleton, Vimentin is widely expressed in various mesenchymal cells and cells derived from the mesoderm [58]. Vimentin immunoreactivity, together with the predominantly fibroblast-like morphology, was consistent with mesenchymal-like characteristics of the NCSB culture, although these observations alone do not establish a definitive cellular lineage.

### 4.2. Effects of TGF-β1 on Collagen-Related Expression and Accumulation in NCSB Cells

As a multifunctional cytokine, TGF-β signaling regulates a broad spectrum of cellular activities, encompassing cell proliferation, apoptosis and differentiation, ECM remodeling and fibrosis. TGF-β1 has been widely used as an experimental stimulus to investigate fibroblast activation and fibrosis-related cellular responses, including changes in collagen-related expression and ECM remodeling [15,16]. Previous studies in *N. coibor* have demonstrated that the TGF-β/Smads signaling pathway participates in proline-associated collagen deposition in the swim bladder, as supported by the inhibitory effects of SIS3 on collagen-related gene expression and collagen deposition [21]. More recently, NcSmad3 was shown to enhance the promoter activities of *col1a1* and *col1a2* in swim bladder cells [22]. These findings provide a biological rationale for selecting TGF-β1 as an experimental stimulus to further evaluate the collagen-related responsiveness of the long-term cultured NCSB cells. It has been reported that recombinant TGF-β protein from *Carassius auratus L.* can induce the growth of goldfish CCL71, and the effect is dose-dependent [59]. In contrast, TGF-β1 downregulates proliferation in primary, non-transformed human lung fibroblasts via the SMAD-dependent pathway [60]. In the present study, we evaluated NCSB cell proliferation following treatment with 0, 5, 10, 20, and 50 ng/mL TGF-β1. Although 10 ng/mL TGF-β1 reduced cell proliferation compared with the control group, the cells maintained continuous growth during the treatment period. These discrepant responses to TGF-β may be attributed to differences in the tissue origin and intrinsic biological characteristics of the cells. For the subsequent experiments, 10 ng/mL TGF-β1 was selected based on concentrations used in previous in vitro studies [48,49] and the proliferation response observed in the present experiment; however, this concentration was not optimized specifically for collagen-related endpoints.

In mammals, elevated TGF-β levels can induce fibroblast activation and their transformation into myofibroblasts, thereby promoting collagen synthesis and deposition [61]. In the present study, treatment of NCSB cells with 10 ng/mL TGF-β1 increased *Col1a1* and *Col1a2* expression and hydroxyproline content in the culture supernatant over the treatment period. α-SMA functions as a classical marker of fibroblast activation. Previous studies have shown that TGF-β stimulation can induce quiescent fibroblasts to upregulate α-SMA expression, acquire enhanced contractility and extracellular matrix synthesis capacity, and thereby undergo myofibroblast-like transformation [61]. Previous studies in fish have also reported TGF-β-associated changes in fibroblast-related characteristics. In *Oncorhynchus mykiss* cardiac fibroblasts, TGF-β1 treatment increased *col1a1* expression and induced changes in α-SMA organization and other cytoskeletal markers [20]. Similarly, TGF-β treatment of *Chrysophrys auratus* tail muscle-derived cells increased *col1a* and *sma* expression and was accompanied by morphological changes and actin bundling [62]. Beyond these changes in collagen-related expression and fibroblast-associated characteristics, studies in human lung fibroblasts have shown that TGF-β-induced collagen protein production requires glutamine availability and its conversion to glutamate [63]. In the present study, TGF-β1 significantly upregulated α-SMA and COL1A1 expression. Together with the increased collagen content estimated from hydroxyproline measurements in the culture supernatant and more intense Sirius Red staining, these findings indicate a coordinated collagen-related response of NCSB cells to TGF-β1. The increase in α-SMA is consistent with a fibroblast activation-associated response, but additional phenotypic and functional markers would be required to establish myofibroblast differentiation. Furthermore, although the increased collagen content in the culture supernatant is consistent with the observed collagen-related response, it does not necessarily indicate an increased rate of collagen biosynthesis or secretion.

Through transcriptomic analysis, we explored the transcriptional responses associated with TGF-β1-induced collagen-related changes in NCSB cells. Treatment with TGF-β1 was associated with differential expression of 1928 genes in the transcriptomic analysis, and these differentially expressed genes were enriched in 20 KEGG pathways, including “TGF-beta signaling pathway”, “ECM-receptor interaction”, “Focal adhesion” and “Cytokine–cytokine receptor interaction”, which are associated with cell signaling regulation and extracellular matrix-related processes. “ECM-receptor interaction” is considered to play a role in maintaining structure and tissue integrity, while “focal adhesion” as an important physical connection between cells and ECM, transmits external mechanical and chemical signals into cells, regulating cell differentiation, proliferation and adhesion [64,65]. These findings suggest that the TGF-β signaling pathway and ECM-related processes may be involved in the transcriptional response of NCSB cells to exogenous TGF-β1 stimulation. However, pathway enrichment alone does not demonstrate the functional activation of these pathways or establish their causal roles in the observed collagen-related changes. These transcriptomic findings provide preliminary insights into the molecular responses of NCSB cells to TGF-β1 and identify candidate pathways for future functional investigation of swim bladder collagen regulation.

#### Limitations of the Study

One limitation of the present study is that the NCSB culture was originally derived from a single fish and a single independent primary culture. Therefore, donor-specific variation could not be assessed, and the observed characteristics may not fully represent swim bladder-derived cells from other individuals of *N. coibor*. In addition, Vimentin expression alone is insufficient to establish the precise cellular lineage of NCSB cells, and additional cell-type-specific markers would be required for further characterization. Moreover, although NCSB cells have been maintained for over 40 passages, their chromosomal characteristics and stability during long-term culture have not yet been evaluated. Future studies should therefore include karyotype analysis to further characterize the genetic features of this cell culture. A further limitation of this study is that RNA from three biological samples was pooled to generate each sequencing library, resulting in only two libraries per treatment group. This design limited the assessment of biological variability and the statistical robustness of the differential expression analysis. Therefore, the transcriptomic findings should be interpreted as exploratory and used primarily to identify candidate genes and pathways for further investigation.

## 5. Conclusions

In this study, we further characterized the long-term cultured NCSB cells derived from the swim bladder of *N. coibor*. NCSB cells exhibited a fibroblast-like morphology and responded to TGF-β1 with increased expression of collagen-related genes and proteins, increased collagen-associated hydroxyproline in the culture supernatant, and more pronounced Sirius Red staining. Therefore, NCSB cells may provide a useful in vitro model for studying collagen regulation in fish. Based on previous applications of NCSB cells, this model may also facilitate future studies on the nutritional regulation of collagen deposition. However, further characterization of the NCSB model and complementary in vivo studies are still needed.

## Figures and Tables

**Table 1:** Primers used in qRT-PCR.

Gene	Forward Primer (5′→3′)	Reverse Primer (5′→3′)
*CTGF*	GCACGGTGTACAAGAGTGGA	CTGGCACCTTGACTCGTCTT
*Col1a1*	AGACCTGCGTGACTCCCA	AGCCCTCGCTGCCATACT
*Col1a2*	CAAGAACAGCGTTGCCTACAT	ACGGAGAAGGTGAAGCGG
*TGFβ*	ATCCCAGTTCCAGCACAAGA	CCAGCCCAGGTCCTTCTTAA
*BMPRI*	ATGGAGGGGAGAAAAGGTGG	GTTCTCGTGGTAGTCGGTGA
*THBS1*	CAACAAGAACGCCTGTCCAA	TACCATTGCCAGTGTAGCCA
*Smad1*	AGGAAGAGAAATGGGCGGAA	ATGCGGCAGACCTTTCCTAT
*Id*	ACAAGAAGGCGAGCAAAGTG	GCACATAATCCTGTCGTCCG
*18s*	AGCTCGTAGTTGGACTTCGG	CGGCCTGCTTTGAACACTCT

## Data Availability

The datasets generated during and/or analyzed during the current study are available from the corresponding author on reasonable request.

## Supplementary Materials

The following supporting information can be downloaded at: https://www.mdpi.com/article/10.3390/ani16203200/s1, Table S1. Summary of established swim bladder-derived cell lines, culture conditions, morphological characteristics and primary research purpose across fish species. Figure S1. Mycoplasma testing. Sample 1: positive control (red); sample 2: negative control (yellow); samples 3–5: culture supernatants collected from three independent cell cultures after 4 days of culture. Figure S2. Gene Ontology (GO) functional classification of differentially expressed genes (DEGs) between control and TGF-β1-treated NCSB cells. The 10 most represented GO terms in each of the three categories—biological process (BP), cellular component (CC), and molecular function (MF)—are presented.

## Abbreviations

The following abbreviations are used in this manuscript:

ECM	Extracellular matrix
TGF-β	Transforming growth factor-β
TGF-β1	Transforming growth factor-β1
(α-SMA)	α-smooth muscle actin
MMPs	Matrix metalloproteinases
PBS	Phosphate buffer solution
DMEM/F12	Dulbecco’s modified Eagle’s medium/Ham’s Nutrient Mixture F12
FBS	Fetal bovine serum
bFGF	Basic fibroblast growth factor
IGF-I	Insulin-like growth factor I
DMEM	Dulbecco’s modified Eagle’s medium
L15	Leibovitz’s L15
COI	Mitochondrial cytochrome c oxidase subunit I gene
CCK-8	Cell Counting Kit-8
PCR	Polymerase chain reaction
GFP	Green fluorescent protein
PFA	Paraformaldehyde Fix Solution
PBST	PBS containing Triton X-100
qRT-PCR	Quantitative real-time PCR
TGFβ	Transforming Growth Factor Beta
BMPRI	Bone Morphogenetic Protein Receptor 1
THBS1	Thrombospondin 1
Smad1	Mothers Against Decapentaplegic Homolog 1
Id	Inhibitor of DNA Binding
Hyp	Hydroxyproline
ECL	Enhanced chemiluminescence detection kit
FPKM	Fragments per kilobase of transcript per million mapped reads
DEGs	Differentially expressed genes
GO	Gene Ontology
KEGG	Kyoto Encyclopedia of Genes and Genomes
SEM	Standard error of the mean
ANOVA	One-way analysis of variance
CTGF	Connective tissue growth factor
