# Supplementary material for: Establishment and Characterization of a Swim Bladder-Derived Cell Line from Chu’s Croaker (Nibea coibor) for Studying Collagen Regulation
Source: Animals (Basel). 2026 Oct 12;16(20):3200. doi: 10.3390/ani16203200 (PMC13700907; doi:10.3390/ani16203200)
Supplement: Supplementary file 1 [file animals-16-03200-s001.zip › animals-4558821-supplementary.pdf]

Supplementary Tables

Table S1. Summary of established swim bladder-derived cell lines, culture conditions, morphological characteristics and primary research purpose across fish species.

Species	Cultivation mode	Culture medium	Serum concentration	Incubation	Morphology	Primary research purpose	Author (time)
Ctenopharyngodon idella	Tissue explant culture	M199	10-20%	32℃	fibroblast-like cells	Virus isolation and study.	Lu (1990) [30]
Labeo rohita	Tissue explant culture	L-15	15%	28℃	epithelial-like cells	Viral disease diagnosis; virus isolation and study.	Lakra (2010) [31]
Carassius auratus	Tissue explant culture	L-15	20%	30℃	fibroblast-like cells	Viral disease diagnosis; virus isolation and study.	Rougée (2007) [32]
Lates calcarifer	Enzymatic dissociation	L-15	10%	28℃	epithelial-like cells	Virus -induced apoptosis	Lai (2008) [33]
Channa striatus	Tissue explant culture	L-15	20%	25℃	fibroblast-like cells	Virus isolation and study.	Zhao (2004) [34]
Epinephelus akaara	Tissue explant culture	L-15	10%	25℃	fibroblast-like cells	Virus–host interaction.	Huang (2009) [35]
Epinephelus awoara	Enzymatic dissociation	L-15	5-15%	28℃	epithelial-like cells	Virus–host interaction; virus isolation and study.	Lai (2003) [36]
Nibea coibor	Tissue explant culture	DMEM/F12	15%	28℃	fibroblast-like cells	collagen regulation	Lin (2020) [11]

The NCSB cells characterized in the present study were derived from the same swim bladder cell resource used in previous studies of *N. coibor*

Supplementary Figures

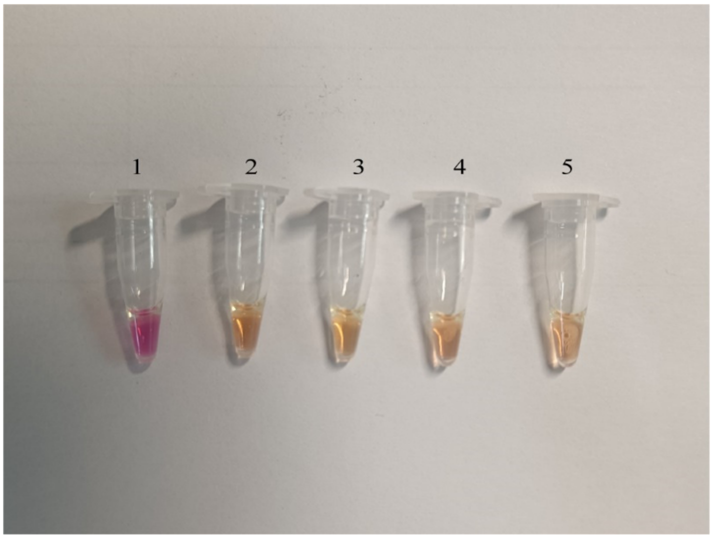

Figure S1. Mycoplasma testing. Sample 1: positive control (red); sample 2: negative control (yellow); samples 3–5: culture supernatants collected from three independent cell cultures after 4 days of culture.

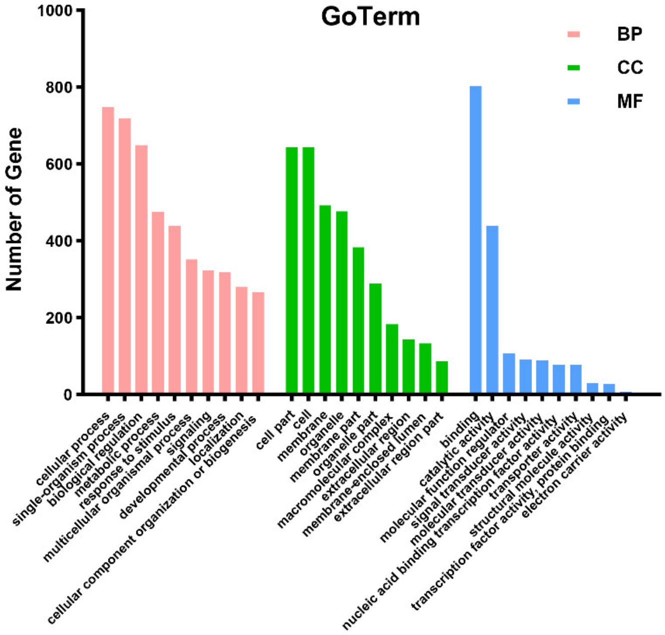

Figure S2. Gene Ontology (GO) functional classification of differentially expressed genes (DEGs) between control and TGF-β1-treated NCSB cells. The 10 most represented GO terms in each of the three categories—biological process (BP), cellular component (CC), and molecular function (MF)—are presented. .
